# Launching a Teaching Academy (Virtually) During a Pandemic: Silver Linings to a Challenge

**DOI:** 10.7759/cureus.37245

**Published:** 2023-04-07

**Authors:** Jennifer Foster, Stuart Goldman, Patrick G Hughes, Vijaya Iragavarapu, Joanna Drowos

**Affiliations:** 1 Department of Medicine, Florida Atlantic University Charles E. Schmidt College of Medicine, Boca Raton, USA; 2 Internal Medicine, Florida Atlantic University Charles E. Schmidt College of Medicine, Boca Raton, USA; 3 Department of Clinical Neuroscience, Florida Atlantic University Charles E. Schmidt College of Medicine, Boca Raton, USA; 4 Department of Emergency Medicine, Florida Atlantic University Charles E. Schmidt College of Medicine, Boca Raton, USA; 5 Department of Biomedical Sciences, Florida Atlantic University Charles E. Schmidt College of Medicine, Boca Raton, USA

**Keywords:** medical education, professional development, covid-19 pandemic, teaching academies, faculty development

## Abstract

The pandemic disrupted our plans to launch a Teaching Academy to formally support medical educators. Moving forward virtually provided a collaborative and supportive network to plan and deliver professional development activities to navigate pandemic challenges. Through sharing and practicing new teaching technologies together, the social connection and engagement with colleagues helped navigate pandemic challenges.

## Editorial

Background

Since their establishment in 2001, teaching academies have supported and advanced educators at a number of colleges of medicine and other institutions. For example, the Academy at Harvard Medical School recognizes teaching contributions, bringing educators together to foster innovation through an idea-sharing forum across departments and focus areas [1]. Based on the success of previous programs, the Charles E. Schmidt College of Medicine at Florida Atlantic University (FAU-COM) created a workgroup in the spring of 2019 to develop a mission statement, criteria, and selection process to launch a Teaching Academy. Applications were distributed in early 2020, with plans for the eight faculty selected for the inaugural class to be inducted in the spring of 2020.

In March 2020, the COVID-19 pandemic resulted in a transition to remote work and virtual medical education. Medical students and faculty were displaced from clinical learning environments and medical school campuses, challenging the delivery of curricula [2]. Faculty were challenged to adapt to virtual teaching and remote work while developing new skills to effectively deliver new models of learning. Faculty development is essential to the success of faculty taking on new roles or teaching models. One known barrier to participation in development activities is competing clinical responsibilities. In this case, the conflict was heightened due to the demand for clinical services related to the pandemic and the lack of supported time for educational pursuits [3]. During the transition to virtual work and teaching, educational leaders at FAU-COM considered whether to move forward with the plans to launch a Teaching Academy or postpone activities until the disruption to academic medicine stabilized.

New expectations and directions

Our College decided to move forward and launch our inaugural class of the Teaching Academy, as we were far along in our planning process. Since COVID-19 limited options for in-person programming, we quickly realized that the same technology we were using to connect with and teach our learners could be leveraged for the faculty group's purpose. Our College quickly adapted multiple platforms for virtual meetings providing options for continued brainstorming and collaboration. As faculty, we were forced to embrace these technologies rapidly for teaching purposes, curricular and departmental meetings, and professional development activities. Virtual meetings allowed faculty at varied clinical sites to participate in development activities without traveling. While group selection had occurred before the pandemic, participation likely increased due to the virtual nature of the academy. Our group spent more time engaged in discussion and sharing resources during our meetings. Additionally, we used technology to engage experts and guest speakers from around the country to deliver programming to a wider audience, without the expense of travel or meeting logistics. This format change reduced barriers for faculty from other sites to participate in activities and allowed for additional programs with national experts without adjusting the allotted budget. We minimized the time commitment due to concerns for burnout, virtual meeting fatigue, and stress, letting members discuss topics where they felt support was needed.

As the pandemic and virtual work continued, the ongoing challenge to identify and collaborate with like-minded educators required unique solutions. With faculty working from home, many experienced a limited connection to learners and colleagues. The Teaching Academy supported discussing pandemic-related challenges at work or home, while members provided support to each other. Additionally, members were experimenting with technology and strategies to engage learners and trying to minimize disruption to clinical learning. Academy meetings provided a forum for conversations and problem-solving. Additional unexpected but welcome benefits included social connections, new relationships, and ultimately laughter and fun at meetings during stressful times.

Additionally, the disruption in clinical learning and classrooms resulted in adapting to new teaching methods and technologies. The Teaching Academy allowed sharing of resources and best practices related to these changes. Members supported each other in discussing challenges around connecting with learners and navigating virtual teaching while meeting curricular goals. In addition to reviewing best practices in virtual teaching and sharing resources, the experience itself became the teacher. While originally delaying the launch until a more stable time seemed appropriate, through flexibility and an emphasis on what faculty needed at the moment in time, we maintained our momentum and demonstrated a need for resources, prioritizing our group as a professional development opportunity during a challenging time for medical school faculty.

Future directions

Assessment of outcomes will be based on the original goals of the Teaching Academy as laid out by the original planning workgroup prior to the COVID-19 pandemic. The original goal of developing teachers was expanded to include connecting faculty during virtual work. Another indicator of success includes the expansion of membership as more faculty see the value of the group and seek membership. The Teaching Academy hosts visiting scholars to provide faculty development around teaching and has engaged the large majority of faculty at the College in these programs. Technology has increased the inclusivity of the group as faculty from a variety of clinical sites can fully participate. Educational scholarship can and will be tracked by group members, compared to output prior to engagement. As far as professional development opportunities and resources to improve skills, the Teaching Academy has offered additional development and recognition opportunities for members in a virtual environment. As the pandemic is fading, future Teaching Academy activities will embrace a hybrid model of live/virtual meetings to optimize the most benefit for all faculty and ultimately for learners. The opportunity for connection that occurs when groups meet in person can be capitalized on for some meetings while reducing the participation barrier for those who are not able to join in person. Some topics and activities are more effective with an in-person format. Finally, in resource-challenged times, embracing a hybrid model will have advantages, sparing both time and money.

In spite of the disruption that followed the COVID-19 pandemic, moving forward with the launch of the Teaching Academy proved an effective continuing professional development tool. Once our group made the commitment to advance, taking advantage of technology, engaging in development specific to pandemic-related teaching, and providing support in navigating the challenges of the pandemic, the value of participating in virtual activities helped faculty overcome traditional barriers to participating in faculty development. In order to maintain momentum and advance the group, future cohorts will also require strong motivation from members, as well as time management skills. Additional opportunities offered will foster educational innovation, promote scholarship and research, and support educators in developing academic skills, avoiding isolation, and navigating the changes in academic medicine.
